# Running the gauntlet: challenges to genome integrity in spermiogenesis

**DOI:** 10.1080/19491034.2024.2339220

**Published:** 2024-04-09

**Authors:** Maiko Kitaoka, Yukiko M. Yamashita

**Affiliations:** a Whitehead Institute for Biomedical Research and Howard Hughes Medical Institute, Cambridge, MA, USA; b Department of Biology, Massachusetts Institute of Technology, Cambridge, MA, USA

**Keywords:** DNA damage, Double stranded breaks (DSB), genome integrity, protamines, Spermiogenesis

## Abstract

Species’ continuity depends on gametogenesis to produce the only cell types that can transmit genetic information across generations. Spermiogenesis, which encompasses post-meiotic, haploid stages of male gametogenesis, is a process that leads to the formation of sperm cells well-known for their motility. Spermiogenesis faces three major challenges. First, after two rounds of meiotic divisions, the genome lacks repair templates (no sister chromatids, no homologous chromosomes), making it incredibly vulnerable to any genomic insults over an extended time (typically days-weeks). Second, the sperm genome becomes transcriptionally silent, making it difficult to respond to new perturbations as spermiogenesis progresses. Third, the histone-to-protamine transition, which is essential to package the sperm genome, counterintuitively involves DNA break formation. How spermiogenesis handles these challenges remains poorly understood. In this review, we discuss each challenge and their intersection with the biology of protamines. Finally, we discuss the implication of protamines in the process of evolution.

## Introduction

The production of healthy gametes through the process of gametogenesis ensures the continuity of lineages across evolutionary timescales, as the germline is the sole cell type that passes genomic information from one generation to the next. Thus, gamete genome quality is paramount. Due to their abundance, sperm cells have largely been considered ‘cheap’ and ‘disposable’ since only one sperm cell is needed for fertilization, while many more do not pass their genome to the next generation. For example, human males can release 30–300 million sperm at a time [1], while *Drosophila* males transfer approximately 1500 sperm per mating [2]. Therefore, sperm selection during fertilization, where only the most fit amongst many contenders will be chosen, is generally assumed to be sufficient to select for a ‘fit genome’ [3,4]. Because of this assumption, it is underappreciated that spermiogenesis itself imposes great risks on the genome integrity of developing sperm cells, and thus mechanisms must exist to protect the germline genome.

**Figure 1.**
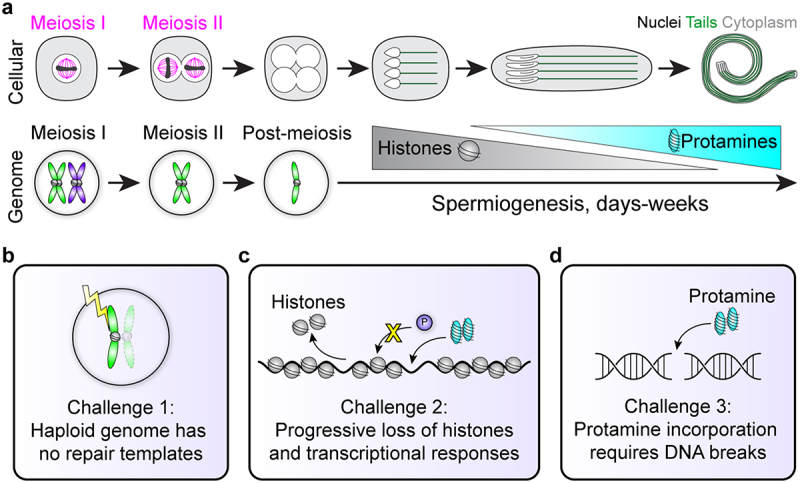
Cellular and genomic changes during spermiogenesis. (a) Sperm undergo dramatic morphological differentiation from round haploid cells after meiosis to elongated nuclei with flagellar tails (top). Meanwhile, the genome first becomes haploid after meiosis, and then changes genomic architecture as histones are exchanged for protamines. Importantly, cells in meiosis I or II have homologous chromosomes or sister chromatids that provide 3 or 1 DNA templates, respectively, for potential DNA repair (bottom). Note that homologous recombination is not depicted in this cartoon. (b-d) Spermiogenesis contains 3 major challenges to genome integrity (see main text).

In the testis, germ cells undergo successive rounds of mitosis, followed by reductive meiotic divisions [5,6]. Spermiogenesis refers to the post-meiotic stages of sperm development that ultimately create mature sperm cells [7]. Uniquely to male gametogenesis, this process of spermiogenesis is extremely long-lasting, taking days in insects and weeks in mammals [6–10], and involves challenges to gamete and genome integrity that are not seen elsewhere in the life cycle of organisms. Developing haploid spermatids must survive a prolonged period of time that encompasses major morphological changes as they become hydrodynamic, terminally differentiated motile cells (Figure 1(a)). The genome’s haploid state makes this period particularly dangerous for gamete genome quality, as there are no homologous chromosomes or sister chromatids that could serve as potential repair templates for homologous recombination (HR)-mediated repair, the most accurate form of DNA repair [11]. This is further complicated by programmed hypercompaction of the sperm genome, mediated by sperm-specific small, nuclear basic proteins (SNBPs) called protamines [7,12]. This genomic reorganization, the histone-to-protamine transition, itself adds complications to sperm genome integrity by silencing transcriptional-level responses and inducing breaks to repackage DNA [13,14] (Figure 1(b-d)). Altogether, spermiogenesis appears to pose obstacles that risk genome integrity and prevent the production of high-quality, undamaged gametes, which is counterintuitive to the evolutionary goal of protecting the germline for generational inheritance.

Here, we review these major challenges during spermiogenesis, with a particular focus on their intersection with protamine biology. We discuss potential cellular solutions and evolutionary rationales that support gamete genome quality despite this fraught differentiation trajectory.

## Challenge 1: lack of repair templates

Spermiogenesis is accompanied by drastic morphological changes to the cell and nucleus to create long flagellar tails and elongated hydrodynamic sperm heads. Post-meiotic differentiation takes a very long time, lasting several days in insects and multiple weeks in mammals, including humans [6–10,15]. After the reductive meiotic divisions, each developing sperm cell contains only one copy of the genome throughout this long-lasting process, so each cell has no homologous chromosomes or sister chromatids that could serve as templates for faithful HR-mediated repair in the event of DNA damage (Figure 1(b)). This dangerously vulnerable genomic state appears counter to the germline’s evolutionary goal to protect the integrity and quality of its gametes for lineage continuity. However, nothing is known about how developing sperm manage this precarious situation, or what happens to sperm nuclei that suffer from DNA damage.

This vulnerable genomic state after meiosis is even more counterintuitive, particularly in light of the robust mechanisms that operate to ensure genome integrity in the germline prior to meiosis. It is well known that germline cells are extremely sensitive to DNA damage compared to somatic cells, likely to uphold the most stringent genome quality [16–18]. Clinical treatments such as radiation therapy and chemotherapy commonly result in infertility, likely due to germ cells’ higher sensitivity to DNA damage [16,19]. This high sensitivity has been partly explained by a unique feature of germ cells: Early male germ cells prior to entry into the meiotic program trigger a unique cell death response upon exposure to DNA damage and double strand break (DSB) formation, mediated by their cellular connections that support rapid signal transduction [20]. This response demonstrates the early germline’s robust ability to mount a DNA damage signaling cascade that includes phosphorylation of a histone variant (γH2Av in *Drosophila*, equivalent to γH2AX in mammals), which also serves as a reliable marker of DSB formation [20–23]. This also facilitates an increased sensitivity of the germline to DNA damage, because upon DSB detection, germ cells kill not only themselves but also their sister cells in pre-meiotic cysts [20], suggesting that any potentially damaged genome within the cyst is eliminated to minimize the chance of a damaged genome progressing further in spermatogenesis.

Importantly, this high sensitivity to DNA damage and the subsequent cellular responses (i.e., cell death) occurs in diploid germ cells. Throughout this diploid period of development, germ cells undergo extensive DNA quality surveillance that eliminates potentially damaged genomes to ensure quality [24–26]. Subsequently however, meiotic divisions create vulnerable haploid germ cells that lack homologous chromosomes or sister chromatids for an extended period of time until fertilization. Thus, post-meiotic cells are dramatically limited in their genome repair capacity. If anything, only non-homologous end joining (NHEJ) mechanisms, which do not require repair templates, would be possible. However, NHEJ is notably error-prone and leads to deleterious chromosomal aberrations, including frequent mutations, insertions, and deletions [27].

**Figure 2.**
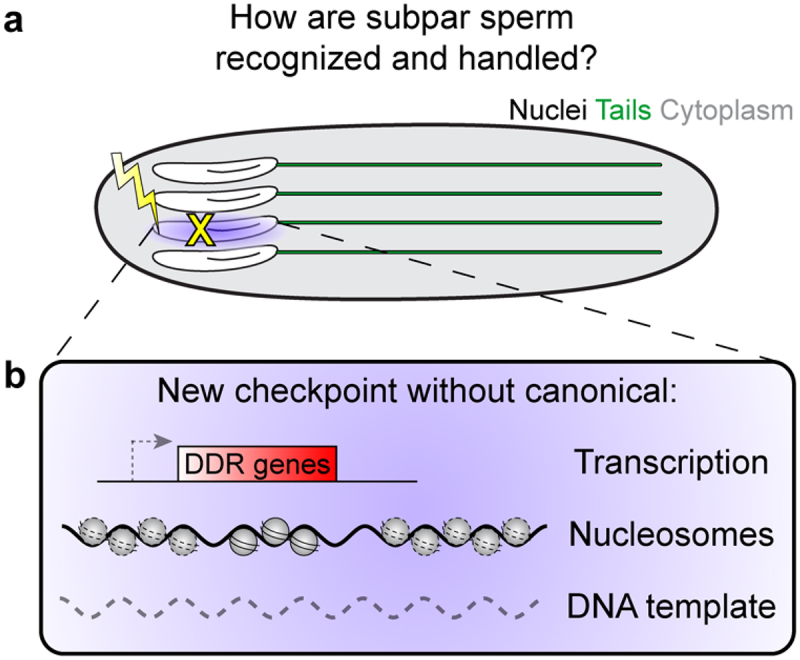
Potential solution to spermiogenesis challenges. (a) If developing sperm are damaged during spermiogenesis, how are they be detected and what is their fate? (b) We propose that a novel checkpoint could exist that does not rely on canonical transcription-based responses, nucleosomes and histone modifications, or a second DNA template.

Thus, the critical puzzle is why the process of spermiogenesis lasts for an extended period of time (days to weeks), while leaving the genome so vulnerable, as if rendering moot the ‘efforts’ of early germ cells to pass only the highest quality, or undamaged, genomes. It is tempting to speculate that spermiogenesis also has mechanisms that prevent harmful mutations from being passed to the next generation, such as through a novel genome quality control checkpoint (Figure 2). This will likely look very different from known cell cycle or other DNA damage response checkpoints due to the haploid genome, as well as the loss of histones and transition to a unique sperm-specific, protamine-wrapped genome architecture, as described below.

## Challenge 2: silent genome, lack of responses

Complicating this vulnerable haploid state further, spermiogenesis is uniquely accompanied by the histone-to-protamine transition [12] (see below). As histones are removed from sperm genomes, transcription shuts down, and post-meiotic spermatids lose canonical chromatin-based transcriptional responses (Figure 1(c)). Reflecting this limited and progressive inability of developing spermatids to transcribe, the spermiogenesis program across species decouples transcription and translation, and the massive protein production required to generate the long sperm tail is achieved by relying on translation of mRNA pools that were transcribed much earlier and stored in a silent state [13,28–30]. Recent single-cell and single-nucleus transcriptomic analysis confirmed that *Drosophila* post-meiotic spermatids transcribe only a limited subset of genes [28,31]. Thus, for most genes, transcription abruptly ceases in later elongating spermatids, coinciding with the histone-to-protamine transition [12,28,32]. Thus, spermiogenesis relies solely on translational and post-transcriptional regulation for functional expression of most proteins needed for sperm maturation. Accordingly, this decoupling dramatically reduces any ability of post-meiotic cells to respond to changing conditions or insults, such as DNA damage, and it becomes impossible for histone-depleted spermatids to respond to DNA damage via canonical pathways involving transcription.

Over many days and weeks as the histone-to-protamine transition continues, the genome progressively loses not only the ability to transcriptionally respond to DNA damage, but also DNA damage surveillance mechanisms that rely on histone modifications to trigger downstream responses (Figure 1(c)). The most notable example is phosphorylation of histone variant H2Av/H2AX, referred to as γH2Av/γH2AX, which recruits downstream effectors to respond and potentially repair DNA [22,23,33]. Once histones are exchanged in spermiogenesis, phosphorylation of a histone variant is no longer an option, and post-meiotic cells may no longer be able to detect DNA damage throughout the entire, long-lasting process. Intercellular signaling also becomes limited as cytoplasm and nucleoplasm volume begin to shrink during the dramatic elongation of both cell and nucleus morphology [7,34] concomitant with the reorganization of the genome. In the case of DNA damage, even if H2Av/H2AX can be phosphorylated, downstream cellular signals could become more difficult to activate. This is in sharp contrast to the high sensitivity to sense and respond to DNA damage in diploid early germ cells through their cellular connections (described above) [20].

Without histones, cells have no way to activate DNA damage responses mediated by transcriptional regulation throughout the many days and weeks of spermiogenesis. It is entirely unknown if and how developing sperm may sense when something has gone awry and a nucleus has become damaged (Figure 2). Given the hypersensitivity to damage that early germ cells have to maintain genome quality, it is difficult to imagine that germ cells undergoing spermiogenesis give up everything (protection, repair, sensing, apoptosis and cell elimination) and let damaged DNA be passed to the next generation. The silent genomic state likely means that there may be no means to repair damaged spermatids, consistent with the limited repair capacity of haploid spermatid genomes. However, this does not preclude the existence of an entirely new strategy to handle subpar sperm, for example through elimination, which has yet to be discovered (see Potential solutions below).

## Challenge 3: reorganization of genome architecture for extreme DNA compaction

The above challenges expose how vulnerable paternal genomes are to external sources during spermiogenesis. As if those were not dangerous enough, spermiogenesis truly tests the limits of genome integrity and quality control as protamines repackage DNA. Though not all organisms use SNBPs, such as zebrafish [35], cnidarians (e.g., jellyfish, corals) [36], and echinoids (e.g., sea urchins) [37], genome remodeling from the nucleosome-based architecture found in all other somatic and germline cell types to a hypercondensed protamine-based architecture is one of the most unique features of sperm cells in many animals. This dramatic reorganization sequentially replaces histones with sperm-specific SNBPs, which hypercompact the DNA from 10X in humans to 200X in *Drosophila* [8,38,39]. Though the precise mechanisms can vary across species, canonical histones are first destabilized by post-translational modifications, notably H4 hyperacetylation, H2A/B ubiquitination, and SUMOylation [12,40,41]. As developing sperm continue to elongate morphologically, transition proteins follow histone removal before protamines are finally incorporated into DNA, where they form toroidal structures of sperm chromatin that persist in mature sperm until fertilization [38,42]. While we still lack a comprehensive, mechanistic understanding of the histone-to-protamine transition, despite its essentiality to spermiogenesis and male fertility, recent technological advances have provided insights into the nuances and surprises of this process.

SNBPs, i.e., transition proteins, protamines, and protamine-like proteins, are a large and poorly conserved group of DNA-binding proteins whose major molecular function is to condense the sperm nucleus. SNBPs have basic lysine and/or arginine cores that lead to a net positive charge that neutralizes the phosphate backbone of the DNA double helix. These electrostatic interactions are thought to be the major driver of protamine-DNA interactions [43]. In mammals, each protamine wraps 10–15 bp of DNA, compared to 147 bp wrapped around nucleosomes [44], and packages 85–95% of the sperm genome into toroidal structures [38], while histones are retained on the remaining 5–10% with a particular enrichment for the promoters of early embryonic developmental genes [45]. Of note, the centromeric histone, CENP-A, is retained across species and provides a template for the maternal machinery to load in the zygote to retain centromere identity and location information [46]. While sperm genome structure, protamine protein sequence and molecular characteristics have species-specific nuances, many consistent patterns emerge when protamines are utilized in sperm nuclei. Loss of protamines and proper protamine processing lead to defects in sperm DNA compaction, resulting in decreased fertility. Many species encode multiple protamines [43,47–49], and a carefully balanced ratio between protamines is key to fertility in these species [50,51]. In humans, the 1:1 ratio between protamines P1 and P2 is critical for fertility, as even a small increase or decrease is associated with infertility [50–52]. In *Drosophila* and mice, Mst77F and P2, respectively, are first translated as a longer polypeptide but must be cleaved for incorporation into DNA [49,53]. Loss of this cleavage prevents complete histone-to-protamine exchange and causes infertility [54]. However, how different protamines interact together to achieve this tight balance and hypercompact the genome remains a mystery. Recent work on mouse P1 suggests that protamines may mediate higher-order DNA compaction dynamics through post-translational modifications, not just electrostatic charge of amino acids [55].

Strikingly, this protamine-mediated hypercompaction of sperm genomic DNA requires active DNA break formation at a time when the genome lacks repair templates and cannot detect or respond to address the broken DNA (Figure 1(d)). DSB formation has been found in post-meiotic cells across species by immunofluorescence of γH2AX and TUNEL labeling of DNA ends [14,40,56–58]. Knockdown of Topoisomerase IIβ (Top2β) in *Tetrahymena* prevented γH2AX formation and DNA fragment accumulation in post-meiotic nuclei, but not meiotic prophase, suggesting that DNA is broken by Topoisomerase IIβ [56], which likely assists to detangle DNA supercoils as histones are removed. In mice, Top2β is localized to elongating spermatid nuclei that are undergoing the histone-to-protamine exchange, and incubation with Top2β inhibitors eliminated TUNEL staining at these stages [57,59], suggesting Top2β’s involvement in protamine incorporation.

The intrinsic creation of DSBs in spermiogenesis appears counter to the germline’s essential goal to promote and protect a high-quality genome for the next generation, since any unresolved or persistent DNA breaks could be immediately inherited. Whereas Top2β would be capable of repairing DSBs that it creates [60,61], it is unknown if there are other sources of DSBs during histone-to-protamine transition. Additionally, transition proteins have been proposed as the repair proteins, as they promote DNA ligation *in vitro* after single-strand breaks [62,63], although transition proteins lack a predicted ligase domain. A second possibility is that the DNA remains damaged in mature sperm, and repair occurs only after fertilization. In fact, maternal factors involved in the error-prone polymerase theta-mediated end joining (TMEJ) pathway facilitate paternal genome repair when *C. elegans* sperm containing high DNA damage fertilizes the egg [64]. Despite their amoeboid rather than flagellated morphology, *C. elegans* sperm have 3 identified putative SNBPs and appear to undergo a histone-to-protamine transition [65,66]. However, maternally-driven paternal genome repair is often incomplete, such that chromosomal aberrations and harmful mutations were retained and affected the F1 progeny’s gametogenesis and thus subsequent generations [64]. Overall, this suggests that programmed DNA damage from the histone-to-protamine transition is more likely repaired in the male prior to fertilization. This is supported in *Drosophila*, where the male’s genotype influenced the transmission of broken vs. repaired chromosomes [67], and hints that the spermiogenesis may be able to sense the presence of broken DNA and prevent its transmission, either through repair or alternative mechanisms. Understanding whether and how sperm DNA is repaired during spermiogenesis or in the early zygote will be crucial to our understanding of the vulnerabilities of the paternal genome and its cell biological and evolutionary implications. Beyond the molecular mechanisms, new questions emerge about how sperm genomes might sense and respond to DNA breaks, programmed or otherwise, and whether or not these genomic risks are useful rather than solely dangerous.

## Potential solutions to spermiogenesis challenges and implication in evolution

As detailed above, spermiogenesis is rife with challenges to the genome integrity of each new developing sperm cell. At present, many open questions and gaps in our understanding remain. These challenges should make it very difficult to complete spermiogenesis faithfully. Therefore, we propose that there may be mechanisms that ensure sperm genome quality, as well as a biological rationale to evolve a system with as many challenges as this. It is believed from protamine mutants that protamine packaging could be protective against genotoxic insults [52,68], thus shielding the paternal genome before fertilization. However, this protection only applies to the final stages of spermiogenesis once protamines have been successfully incorporated and does not support earlier stages that are actively exchanging histones and protamines. As we speculated above (Challenges 1 and 2), it is unlikely that germ cells undergo the long-lasting spermiogenesis program without any additional protective mechanisms, thereby nullifying all efforts that pre-meiotic cells put in place to sense and eliminate damaged germline genomes. Instead, it is sensible to hypothesize the presence of unique surveillance and defense mechanisms that can sense and repair/remove damaged or otherwise subpar sperm. The genomic and transcriptional capacity of spermatids poses significant limitations, so these mechanisms are likely entirely unique to spermiogenesis and currently remain undiscovered (Figure 2).

The third challenge, where DSB formation is required for protamine-based packaging, could potentially be solved by adding ‘breakable DNA’ to the genome, or locations where a DSB is less deleterious even if the exact original sequence is slightly changed. It appears that DSBs are formed at specific places during the histone-to-protamine transition to avoid mutating essential genetic elements. The sperm ‘breakome’ has recently been described in mouse spermiogenesis [58,69]. DSBs are created across 1.5% of the mouse genome in elongating spermatids. Breaks are associated with purine-pyrimidine repeats of alternating A or T residues, and DSB hotspots appeared at intergenic regions and were enriched on the Y chromosome, which is notably gene-poor and highly repetitive [58,69]. Taken together, DSB formation is likely a programmed component of post-meiotic genome reorganization that occurs mostly in repetitive or non-coding regions of the genome. The location of DSBs is particularly intriguing. In light of the dangerous strategy where cells must break the gamete genome in order to incorporate protamines, perhaps non-coding, repetitive regions that have long been considered ‘junk DNA’ [70], such as satellite DNA, are an ideal place for DSBs. Any mutations that result from the lack of repair templates and machinery would likely be tolerated better at repetitive DNA, rather than at genic regions. Alternatively, intrinsically more fragile AT-rich repetitive sites [71] could be co-opted for this purpose as well. Future investigations into the ‘breakome’ could shed new light on the potential function of satellite DNA as a protective measure against the accumulation of deleterious mutations during the histone-to-protamine transition.

**Figure 3.**
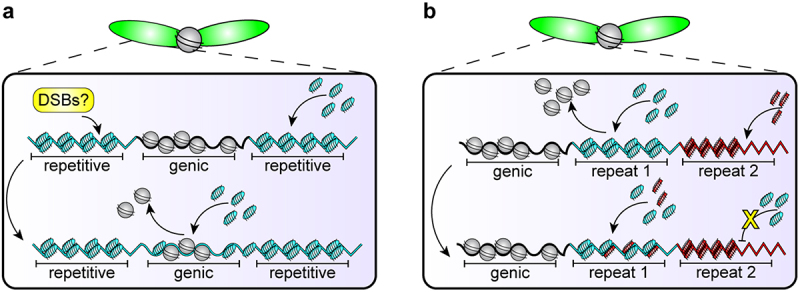
Hypothetical example of co-evolution between DNA sequences and protamines. (a) Perhaps protamines have preferential affinity for repetitive sequences, which could provide a signal to create DSBs safely at repetitive sequences so that genic sequences are not mutated but can incorporate protamines. (b) This preferential affinity could evolve to be sequence-specific such that the ‘wrong’ protamine-DNA pair cannot compact, causing interference with and ultimately failure of the histone-to-protamine transition.

Forming DSBs at ‘safe’ DNA sequences, such as repetitive satellite DNA, for the histone-to-protamine transition has interesting cell biological and evolutionary implications. To do this, protamine genes may need to evolve adaptively to have differential affinity to repetitive vs. genic DNA. If protamines have DNA sequence preference, they could be incorporated in a certain temporal order that differentiates repetitive vs. genic DNA (e.g. repetitive DNA first, followed by genic DNA or vice versa) (Figure 3(a)). In such a scenario, there will be a time window, during which repetitive vs. genic DNA have very distinct chromatin states (e.g. one packaged by histones, and the other by protamines), which can be utilized to target DSBs to non-genic regions (Figure 3(a)).

While protamines are generally considered to be sequence nonspecific, most of these data stem from largely *in vitro* studies that used bulk naked DNA [39,43,72,73]. We currently do not understand the dynamics of protamine incorporation based on DNA sequence, and it remains unknown if some DNA sequences may incorporate protamines faster or slower than others. Interestingly, protamines and other SNBPs are rapidly evolving and have very poor sequence conservation, despite performing critical, conserved functions essential to male fertility (described above) [47]. Combined with the well-known rapid evolution of repetitive DNA sequences between even closely-related species [74–76], it is tempting to speculate that protamines might co-evolve with repetitive DNA to allow for DNA breaks at ‘safe’ DNA sequences during the histone-to-protamine transition.

## Protamines’ connections to meiotic drive

In the light of the potential co-evolution between repetitive DNA and protamines, it is interesting to note that protamines are implicated in the process of meiotic drive. Meiotic drive occurs when a selfish genetic element biases, or drives, its own transmission to the next generation at the expense of organismal fitness [77]. There are 3 major examples of male meiotic drive systems discovered to date in *Drosophila* species in which protamines or the overall histone-to-protamine transition are affected. All three result in abnormal sperm nuclei, defective in compaction, that are subsequently rendered nonfunctional. Firstly, in the *D. simulans* Winters system, the hairpin RNA suppressor locus *Not much yang* (*Nmy*) targets *Distorter on X* (*Dox*) [78–80], which contains homology to the DNA-binding, high mobility group (HMG) domain characteristic of Protamine A/B and other SNBPs [81–83]. Though *Dox* expression does not prevent protamine incorporation post-meiotically [80], Y chromosome-bearing sperm nuclei in *nmy* mutants fail nuclear condensation, leading to predominantly female progeny [78,80]. Secondly, Mst77Y, a Y-linked, multi-copy protamine variant, was recently discovered to interfere with the histone-to-protamine transition in *D. melanogaster* [84–86]. Overexpression of the variant Mst77Y leads to decreased incorporation of Protamine A/B and Mst77F as well as subsequent nuclear decompaction of X chromosome-bearing sperm that skews the resulting progeny’s sex ratio [86]. Lastly, the *Segregation Distorter* (*SD*) system, the most well-known meiotic drive system in *D. melanogaster*, consists of one autosomal locus that encodes both the driver and target: The driver, *Sd*, encodes a truncated duplication of RanGAP, while its target, *Responder* (*Rsp*), are pericentromeric AT-rich satellite DNA repeats whose copy number corresponds to the severity of drive [87–89]. Ultrastructure and cytological studies have pointed to a failure in the histone-to-protamine transition such that wildtype *SD+* sperm nuclei suffer compromised protamine incorporation and nuclear compaction [89–94]. Supporting this, knockdown of Protamine A/B in an *SD* genetic background exacerbates drive [94]. Interestingly, each meiotic drive system adds evidence to a potential compaction-based DNA quality control checkpoint at the very end of spermiogenesis which ultimately eliminates the targeted nonfunctional sperm nuclei.

We lack a deeper understanding that explains why protamine biology is involved in meiotic drive. Indeed, we still don’t know the targets of some systems (i.e., Mst77Y), and the precise cellular mechanisms at play between driver and target remain elusive. However, could this association of protamines with meiotic drive be explained by the potential sequence specificity of protamines? As we speculated in the previous section, protamines may evolve greater sequence affinity for certain DNA sequences in order to generate DSBs preferentially at repetitive DNA during the histone-to-protamine transition (Figure 3(a)). Once established, preferential protamine-DNA affinity could become exploited to disrupt the histone-to-protamine transition at specific repetitive DNA, leading to the demise of specific chromosomes that harbor the targeted repetitive DNA sequence (Figure 3b). Further studies in protamine biology and meiotic drive could reveal how selfish elements take advantage of sperm selection mechanisms, and more broadly, the histone-to-protamine transition as a prime setting for these evolutionary dynamics.

## Future outlooks and conclusions

Highly vulnerable haploid, silent spermatids ought to have some cellular protections and reinforcements to navigate the challenging and protracted spermiogenesis program. A spermiogenesis checkpoint has been proposed for quite some time, particularly in the *Drosophila* system [5,49,91,95–97]. Yet to date, there is no evidence of an active checkpoint or other potential mechanism that surveils spermatid genome quality as sperm undergo this protracted development period that involves dramatic histone-to-protamine genome reorganization. Despite this, it’s tempting to speculate that protamines may be a critical component of DNA quality control mechanisms in spermiogenesis that closely monitor the compaction state of developing sperm. The vulnerable genomic state that lacks potential repair templates dictates that some kind of post-meiotic genome quality surveillance is likely necessary to ensure the continued production and transmission of high-quality sperm. Protamine biology connects to fundamental genome organization, reproductive success, and evolutionary drive systems, implying that protamines could be a pivotal molecular player to ensure not only high-quality genome inheritance but also a means of evolutionary innovation.

## Data Availability

Data sharing is not applicable to this article as no new data were created or analyzed in this study.
